# Supplementary material for: Enhanced photocatalytic dye degradation and hydrogen production ability of Bi25FeO40-rGO nanocomposite and mechanism insight
Source: Sci Rep. 2018 Jul 23;8:11090. doi: 10.1038/s41598-018-29402-w (PMC6056507; doi:10.1038/s41598-018-29402-w)
Supplement: Supplementary file 1 — Supplementary Information [file 41598_2018_29402_MOESM1_ESM.pdf]

Enhanced photocatalytic dye degradation and hydrogen production ability of $Bi_{25}FeO_{40}$-rGO nanocomposite and mechanism insight

M. A. Basith[1,*], Ragib Ahsan[1], Ishrat Zarin[1], and M. A. Jalil[1]

[1]Nanotechnology Research Laboratory, Department of Physics, Bangladesh University of Engineering and Technology, Dhaka-1205, Bangladesh
[*]mabasith@phy.buet.ac.bd

ABSTRACT

A comprehensive comparison between $Bi_{25}FeO_{40}$-reduced graphene oxide(rGO) nanocomposite and $BiFeO_3$-rGO nanocomposite has been performed to investigate their photocatalytic abilities in degradation of Rhodamine B dye and generation of hydrogen by water-splitting. The hydrothermal technique adapted for synthesis of the nanocomposites provides a versatile temperature-controlled phase selection between sillenite $Bi_{25}FeO_{40}$ and perovskite $BiFeO_3$. Both sillenite and perovskite structured nanocomposites are stable and exhibit considerably higher photocatalytic degradation over pure $BiFeO_3$ nanoparticles and commercially available Degussa P25 titania. Notably, $Bi_{25}FeO_{40}$-rGO nanocomposite has demonstrated superior photocatalytic ability as well as stability under visible light irradiation than that of $BiFeO_3$-rGO nanocomposite. The possible mechanism behind the superior photocatalytic performance of $Bi_{25}FeO_{40}$-rGO nanocomposite has been critically discussed.

Electronic Supplementary Information

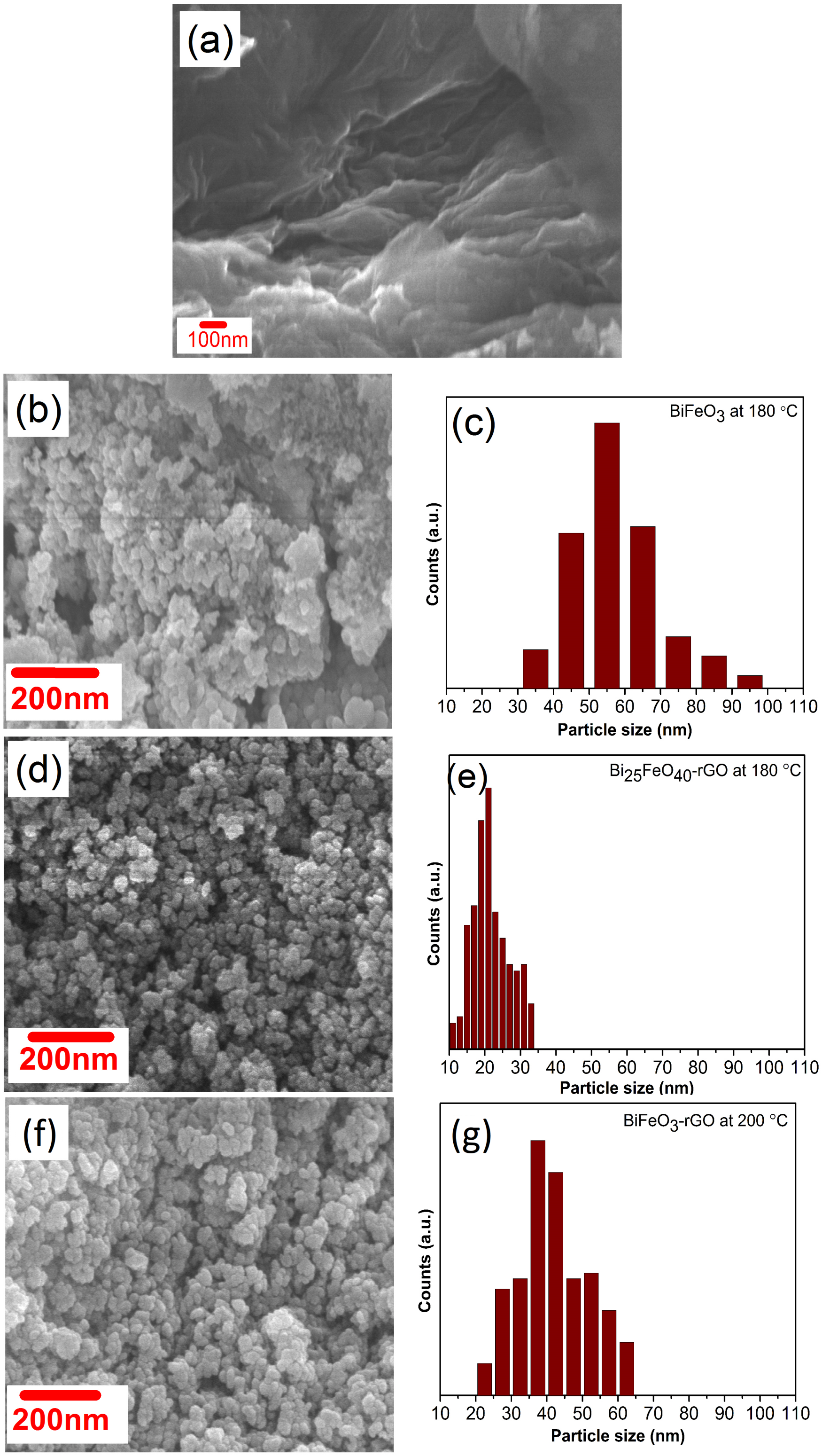

Fig. S1 FESEM images of (a) sheet like structure of GO, (b) perovskite $BiFeO_3$ nanoparticles (c) corresponding histogram of particle size for (b), (d) sillenite $Bi_{25}FeO_{40}$-rGO nanocomposite, (e) corresponding histogram of particle size for (d), and (f) perovskite $BiFeO_3$-rGO nanocomposite; corresponding histogram of particle size for (f) is given in (g) [1]

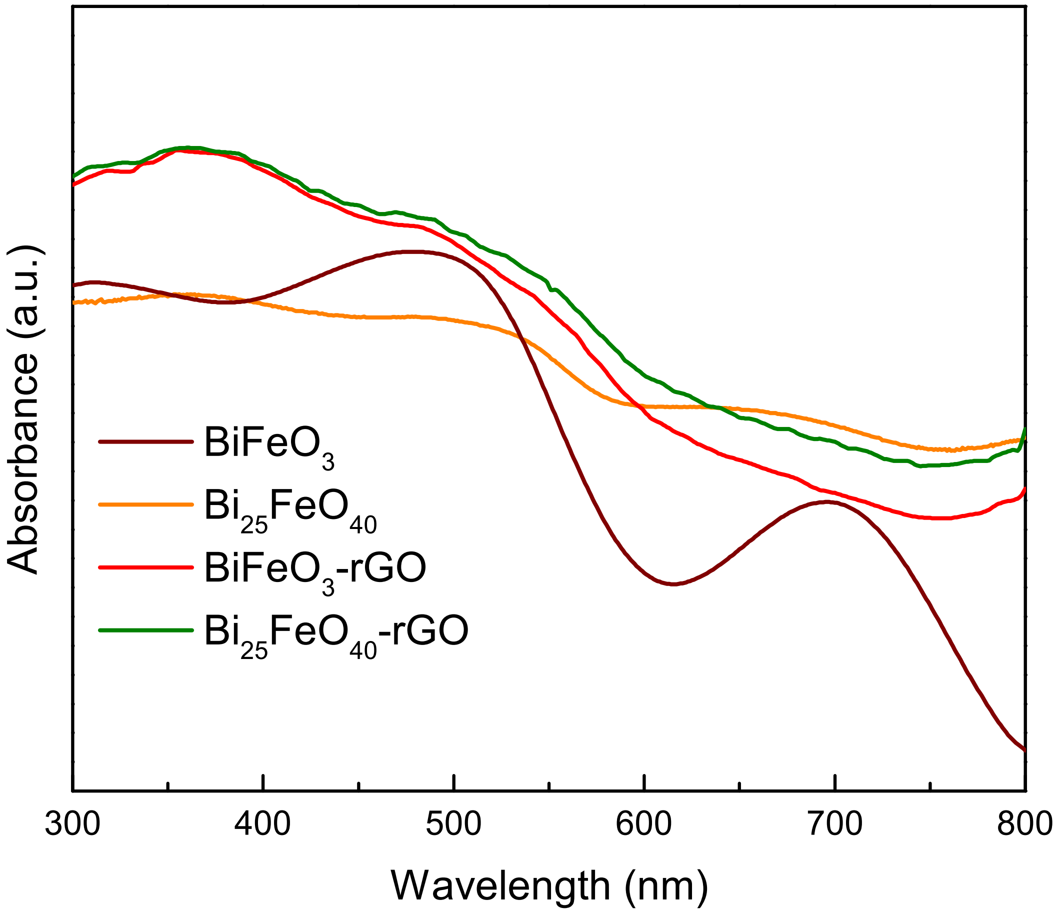

Fig. S2 UV-vis absorbance spectra of $BiFeO_3$ nanoparticles, perovskite $BiFeO_3$-rGO, and sillenite $Bi_{25}FeO_{40}$-rGO nanocomposites

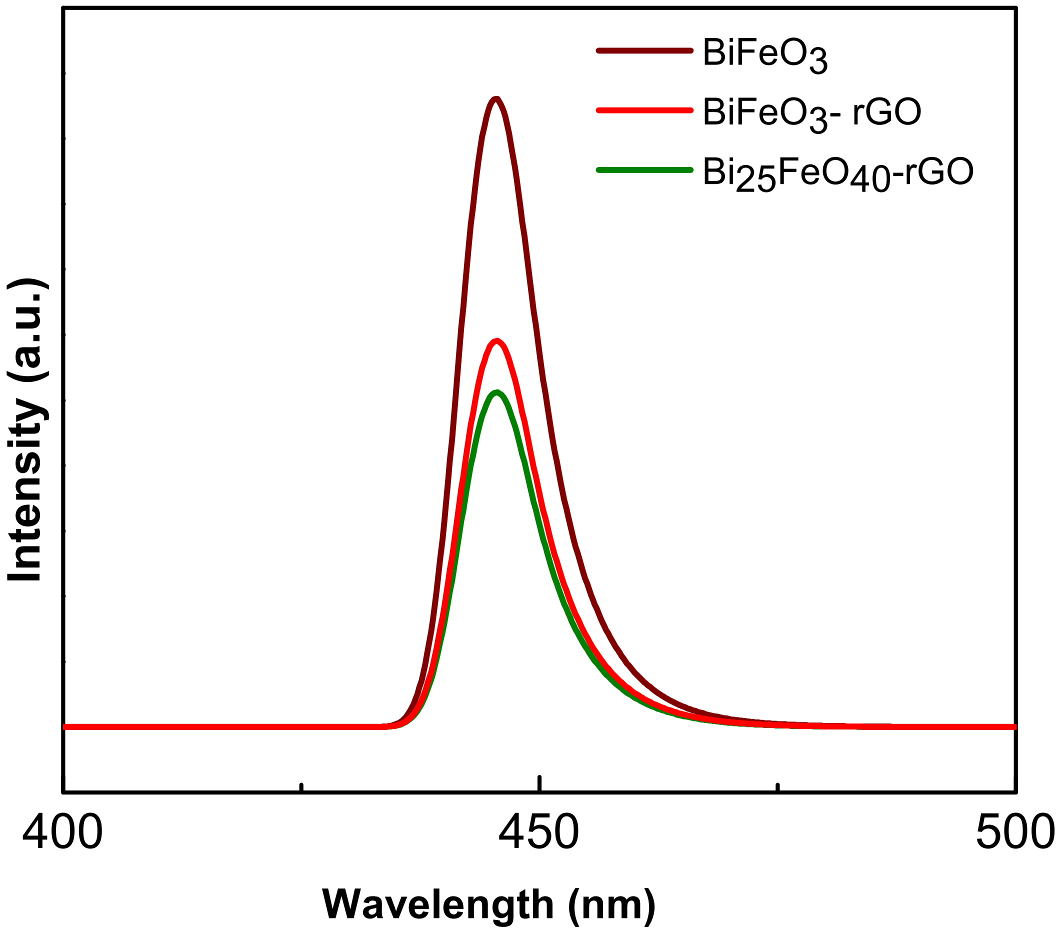

Fig. S3 Steady state PL spectra of $BiFeO_3$ nanoparticles, $BiFeO_3$-rGO nanocomposite and $Bi_{25}FeO_{40}$-rGO nanocomposite

Reference

1. Jalil, M. A. et al. Temperature-dependent phase transition and comparative investigation on enhanced magnetic and optical properties between sillenite and perovskite bismuth ferrite-rGO nanocomposites. Journal of Applied Physics 122, 084902, doi:10.1063/1.4985840 (2017).
